# The Impact of Marital Transitions on Vegetable Intake in Middle-aged and Older Japanese Adults: A 5-year Longitudinal Study

**DOI:** 10.2188/jea.JE20200343

**Published:** 2022-02-05

**Authors:** Taiji Noguchi, Fumi Kondo, Takeshi Nishiyama, Takahiro Otani, Hiroko Nakagawa-Senda, Miki Watanabe, Nahomi Imaeda, Chiho Goto, Akihiro Hosono, Kiyoshi Shibata, Hiroyuki Kamishima, Akane Nogimura, Kenji Nagaya, Tamaki Yamada, Sadao Suzuki

**Affiliations:** 1 Department of Social Science, Center for Gerontology and Social Science, National Center for Geriatrics and Gerontology, Aichi, Japan; 2 Department of Public Health, Nagoya City University Graduate School of Medical Sciences, Aichi, Japan; 3 Department of Nutrition, Faculty of Wellness, Shigakkan University, Aichi, Japan; 4 Department of Health and Nutrition, Faculty of Health and Human Life, Nagoya Bunri University, Aichi, Japan; 5 Atsuta Public Health Center, City of Nagoya, Aichi, Japan; 6 Department of Health and Nutritional Sciences, Nagoya Keizai University, Aichi, Japan; 7 Department of Home Economics, Aichi Gakusen University, Aichi, Japan; 8 Department of Anesthesiology and Intensive Care Medicine, Nagoya City University Graduate School of Medical Sciences, Aichi, Japan; 9 Okazaki Public Health Center, Okazaki Medical Association, Aichi, Japan

**Keywords:** longitudinal study, marital transition, middle-aged and older adults, vegetable intake

## Abstract

**Background:**

Marital transitions are associated with adverse health events, such as mortality and cardiovascular disease. Since marital transitions (eg, becoming widowed) are unavoidable life events, it is necessary to identify modifiable intermediate outcomes. Thus, we examined the association between marital transitions and vegetable intake among middle-aged and older Japanese adults.

**Methods:**

This longitudinal study included Japanese adults aged 40–79 years who received an annual health checkup between 2007 and 2011 (baseline) and 5 years later (follow-up). Marital transitions were classified as whether and what type of transition occurred during the 5-year period and comprised five groups: consistently married, married to widowed, married to divorced, not married to married, and remained not married. Changes in total vegetable, green and yellow vegetable, and light-colored vegetable intake from baseline to follow-up were calculated using the Food Frequency Questionnaire.

**Results:**

Data from 4,813 participants were analyzed (mean age, 59.4 years; 44.1% women). Regarding marital transitions, 3,960 participants were classified as “consistently married,” 135 as “married to widowed,” 40 as “married to divorced,” 60 as “not married to married,” and 529 as “remained not married.” Multivariable linear regression analysis revealed that compared to consistently married, married to widowed was inversely associated with the change in total vegetable intake (β = −16.64, *SE* = 7.68, *P* = 0.030) and light-colored vegetable intake (β = −11.46, *SE* = 4.33, *P* = 0.008).

**Conclusion:**

Our findings suggest that being widowed could result in a reduced intake of vegetables. Hence, dietary counseling according to marital situation is necessary.

## INTRODUCTION

Marital status is an important determinant of an individual’s health.^1^ Married people have been consistently reported to be healthier than people who are not married.^2^^–^^4^ These associations can be attributed to marital protection, such as the benefits of marital ties on health.^5^^,^^6^

There is a large body of evidence on the adverse health effects of marital transitions (ie, marital status changes during a given period). Individuals who have experienced marital transition due to death or divorce have an increased risk of mortality,^7^^,^^8^ cardiovascular disease,^9^ and cognitive impairment.^10^ Potential mechanisms for these adverse health effects include increased unhealthy behaviors,^11^^–^^13^ poorer psychological state,^14^ and reduced social support and social networks.^6^^,^^15^ In particular, because widowhood and divorce among middle-aged and older adults are unavoidable life events, it is crucial to clarify the effects of marital transitions on modifiable intermediate outcomes in order to prevent adverse health events.

Several health-related behaviors that are modifiable and associated with the adverse effects of marital transitions, such as smoking and alcohol intake, have been reported.^11^^,^^12^^,^^16^^,^^17^ In addition, research indicates that marital transitions affect vegetable intake.^11^^–^^13^ In fact, longitudinal studies in the United States have shown that becoming widowed or divorced is associated with reduced vegetable intake in men^11^ and women.^12^ Similarly, a longitudinal study in the United Kingdom reported a reduction in the quantity and variety of vegetables eaten among widowed or divorced men.^13^ Lack of vegetable intake has been found to be associated with mortality due to cardiovascular disease^18^^,^^19^ and cancer.^19^ As such, interventions that address vegetable intake may be advantageous as an approach to minimize the adverse effects of marital transitions.

While the impact of marital transitions on vegetable intake has been investigated in longitudinal studies in Western countries, no such studies have been conducted in East Asia. Given the gender roles in marriage and the dietary habits that differ between cultures, there is a need for research regarding the effects of marital transitions on vegetable intake among East Asians. Because of the historical and cultural background of familism and gender specialization in East Asian societies, there is an expectation that women, more so than men, will perform household tasks, including food preparation.^20^^,^^21^ Thus, marital transitions may have a different impact on men and women in East Asia than in Western countries because of cultural differences in gender specialization. For example, the traditional dietary pattern in Japan is composed of high consumption of soybean products, fish, vegetables, and green tea.^22^^,^^23^ Because the Japanese have a distinctive dietary pattern that is different from Western countries, it is not clear whether the decrease in vegetable intake due to marital transitions applies to Japanese adults. Therefore, the present study aimed to longitudinally examine the association between marital transitions and vegetable intake in middle-aged and older Japanese adults.

## METHODS

### Study population

The present longitudinal study was conducted as a part of the Japan Multi-Institutional Collaborative Cohort Study (J-MICC Study). The J-MICC Study was initiated in 2005 with the aim of obtaining fundamental data for the prevention of lifestyle-related diseases, particularly cancer.^24^^,^^25^ In our study, we recruited 7,098 middle-aged and older adults (aged 40–79 years) between 2007 and 2011 from the J-MICC Study Okazaki area at the Okazaki Public Health Center, Okazaki, Aichi, Japan. During this period, a self-administered health and lifestyle questionnaire was mailed to potential participants before their annual health checkup at the Okazaki Public Health Center. Participants submitted completed questionnaires when they visited the Center. We conducted a follow-up survey 5 years later, between 2013 and 2017. Some participants who received the health checkup submitted the follow-up questionnaire as was done in the baseline survey, whereas those who did not come for the health checkup during the follow-up period submitted the questionnaire by mail. Of the total participants, 5,022 individuals completed the follow-up survey (follow-up rate: 70.8%) and were included in the final analysis.

All participants provided written informed consent, and the study protocol was approved by the ethics committee of Nagoya City University Graduate School of Medical Sciences (No. 70-00-0058). The study was conducted in accordance with the guidelines of the Declaration of Helsinki.

### Vegetable intake

At both baseline and follow-up, vegetable intake was assessed using the semi-quantitative Food Frequency Questionnaire (FFQ), which was developed and validated by the Department of Public Health, Nagoya City University Graduate School of Medical Science.^26^^–^^30^ Participants were asked how often, on average, they consumed 47 foods and beverages over the previous year. Of those, we utilized items on vegetables, not including vegetable juice. There were eight possible responses: “never/rarely,” “1 to 3 times per month,” “1 to 2 times per week,” “3 to 4 times per week,” “5 to 6 times per week,” “once per day,” “twice per day,” and “3 or more times per day.” The average daily consumption of vegetables (g/day) was calculated by weighting typical/standard values (portion size) by frequency of consumption, based on an established method.^27^ We evaluated green and yellow vegetables (eg, pumpkin, carrots, broccoli, and green leafy vegetables), light-colored vegetables (eg, cabbage, daikon radish, shredded dried daikon radish, and burdock/bamboo shoots), and their combined total vegetable intake. “Change in vegetable intake” was the outcome variable and was determined by calculating the difference in intake from the baseline to the follow-up survey. The estimates of vegetable intake were adjusted via residual methods using the daily total energy intake assessed with the FFQ.^31^ However, based on the recommendations of a previous study, 209 participants were excluded because their total energy intake at each survey was either below 600 kcal/day or above 3,500 kcal/day, due to over- or under-reporting their dietary intake.^30^

### Marital transitions

Participants reported their current marital status at baseline and follow-up as married, widowed, divorced, or unmarried. Marital transitions were classified based on whether and what type of transition occurred during the 5-year period and comprised the following five groups: “consistently married” (married at both baseline and follow-up), “married to widowed” (married at baseline, widowed at follow-up), “married to divorced” (married at baseline, divorced at follow-up), “not married to married” (widowed/divorced/unmarried at baseline, married at follow-up), and “remained not married” (widowed/divorced/unmarried at both baseline and follow-up). Thus, there were three groups in which there was marital transition and two groups in which marital status remained unchanged.

### Covariates

Sociodemographic characteristics and health behaviors were assessed using a self-report questionnaire and were used as covariates based on previous studies.^11^^,^^12^ The questionnaire included questions on age, gender, body mass index (BMI), living arrangement, education, employment status, present illness, and lifestyle. BMI was dichotomized as follows: <25.0 or ≥25.0 kg/m^2^. Living arrangement was categorized as living alone, living with spouse, or living with two or more generations. Education was categorized as ≤9, 10 to 12, or ≥13 years. Employment status was categorized as regular employee, non-regular employee, unemployed, or other. Present illness was assessed using a question that asked respondents whether they had received a diagnosis of cancer, heart disease, stroke, hypertension, dyslipidemia, or diabetes. Respondents indicated “yes” or “no” in response to the illness items. Lifestyle was assessed based on drinking, smoking, and walking time. Responses for drinking and smoking were dichotomized as “never/past” or “current.” Walking time was dichotomized as <60 or ≥60 min/day.

### Statistical analysis

First, descriptive statistics were calculated to summarize participants’ characteristics at baseline. Also, the characteristics were compared by marital transitions using ANOVA for age and chi-square tests for all variables except age. Second, we calculated descriptive statistics to summarize vegetable intake according to the five marital transition categories. Third, in order to examine the association between marital transition category and change in vegetable intake, we applied multivariable linear regression analysis and obtained unstandardized regression coefficients (βs) and standard errors (SEs) on the change in vegetable intake. In the analysis, all groups except for “not married to married” were estimated using “consistently married” as a reference group, while “not married to married” was estimated using “remained not married” as a reference group, as based on previous studies.^11^^,^^12^ Three analytical models were created with change in total vegetable intake, change in green and yellow vegetable intake, and change in light-colored vegetable intake as the dependent variables. The models were adjusted for age and gender and for all covariates. To mitigate potential bias caused by missing information, we used the multiple imputation approach under the missing at random (MAR) assumption (ie, that the missing data mechanism depends only on the observed variables). We generated 10 imputed datasets using the multiple imputation by chained equations (MICE) procedure and pooled the results using the standard Rubin’s rule.^32^ In addition, we conducted a gender-stratified analysis. A complete case analysis was also performed as a sensitivity analysis. The significance level was set at *P* < 0.05. We used R software (Version 3.6.3 for Windows; R Foundation for Statistical Computing, Vienna, Austria) for all statistical analyses. The multiple imputation approach used the MICE function (mice package).

## RESULTS

A total of 4,813 participants were analyzed. Participants’ characteristics at baseline are presented in Table 1. Their overall mean age was 59.4 (standard deviation [SD], 9.2) years, and 2,122 (44.1%) of them were women. At baseline, 4,313 (87.5%) participants were married, of which 135 (3.2%) became widowed and 40 (1.0%) became divorced within 5 years. Of those who were not married at baseline, 60 (10.0%) were married within 5 years. In addition, those who were widowed at baseline were older, more likely to be women, and less likely to be living with more than generations. They were also more likely to have a lower level of education, be unemployed, and be non-drinkers and non-smokers. In contrast, those who were not married at baseline and became married were more likely to be obese at baseline.

**Table 1.** Baseline characteristics of the participants

		Marital transitions						*P*-value^a^

		Consistently married	Married to widowed	Married to divorced	Not married to married	Remained not married	Missing	

		*n* = 3,960	*n* = 135	*n* = 40	*n* = 60	*n* = 529	*n* = 89	
Age, years, mean (SD)		59.2 (9.1)	63.6 (7.7)	54.8 (9.3)	56.5 (10.0)	59.4 (10.1)	64.8 (8.5)	<0.001
Gender, *n* (%)	Men	2,332 (58.9)	50 (37.0)	21 (52.5)	33 (55.0)	198 (37.4)	57 (64.0)	<0.001
	Women	1,628 (41.1)	85 (63.0)	19 (47.5)	27 (45.0)	331 (62.6)	32 (36.0)	
Body mass index, kg/m^2^, *n* (%)	<25.0	3,109 (78.5)	106 (78.5)	33 (82.5)	37 (61.7)	416 (78.6)	67 (75.3)	0.034
	≥25.0	847 (21.4)	29 (21.5)	7 (17.5)	23 (38.3)	113 (21.4)	22 (24.7)	
	Missing	4 (0.1)	0 (0.0)	0 (0.0)	0 (0.0)	0 (0.0)	0 (0.0)	
Living arrangement, *n* (%)	Living alone	18 (0.5)	2 (1.5)	4 (10.0)	19 (31.7)	222 (42.0)	3 (3.4)	<0.001
	Living with spouse	1,406 (35.5)	64 (47.4)	7 (17.5)	4 (6.7)	1 (0.2)	37 (41.6)	
	Living with two or more generations	2,536 (64.0)	69 (51.1)	29 (72.5)	37 (61.7)	306 (57.8)	49 (55.1)	
Education (years), *n* (%)	≤9	489 (12.3)	28 (20.7)	5 (12.5)	9 (15.0)	101 (19.1)	24 (27.0)	<0.001
	10 to 12	1,734 (43.8)	64 (47.4)	13 (32.5)	24 (40.0)	220 (41.6)	41 (46.1)	
	≥13	1,737 (43.9)	43 (31.9)	22 (55.0)	27 (45.0)	207 (39.1)	24 (27.0)	
	Missing	0 (0.0)	0 (0.0)	0 (0.0)	0 (0.0)	1 (0.2)	0 (0.0)	0.004
Employment status, *n* (%)	Regular employment	1,604 (40.5)	36 (26.7)	18 (45.0)	34 (56.7)	211 (39.9)	18 (20.2)	
	Non regular employment	680 (17.2)	22 (16.3)	11 (27.5)	11 (18.3)	95 (18.0)	21 (23.6)	
	Unemployed	1,514 (38.2)	67 (49.6)	10 (25.0)	15 (25.0)	203 (38.4)	42 (47.2)	
	Other	161 (4.1)	10 (7.4)	1 (2.5)	0 (0.0)	18 (3.4)	8 (9.0)	
	Missing	1 (0.0)	0 (0.0)	0 (0.0)	0 (0.0)	2 (0.4)	0 (0.0)	
Cancer, *n* (%)	No	3,879 (98.0)	128 (94.8)	40 (100.0)	58 (96.7)	515 (97.4)	87 (97.8)	0.511
	Yes	76 (1.9)	5 (3.7)	0 (0.0)	1 (1.7)	12 (2.3)	2 (2.2)	
	Missing	5 (0.1)	2 (1.5)	0 (0.0)	1 (1.7)	2 (0.4)	0 (0.0)	
Heart disease, *n* (%)	No	3,858 (97.4)	132 (97.8)	40 (100.0)	57 (95.0)	522 (98.7)	87 (97.8)	0.217
	Yes	102 (2.6)	3 (2.2)	0 (0.0)	3 (5.0)	7 (1.3)	2 (2.2)	
Stroke, *n* (%)	No	3,928 (99.2)	134 (99.3)	40 (100.0)	58 (96.7)	525 (99.2)	88 (98.9)	0.287
	Yes	32 (0.8)	1 (0.7)	0 (0.0)	2 (3.3)	4 (0.8)	1 (1.1)	
Hypertension, *n* (%)	No	3,011 (76.0)	102 (75.6)	33 (82.5)	51 (85.0)	404 (76.4)	59 (66.3)	0.477
	Yes	947 (23.9)	33 (24.4)	7 (17.5)	9 (15.0)	125 (23.6)	30 (33.7)	
	Missing	2 (0.1)	0 (0.0)	0 (0.0)	0 (0.0)	0 (0.0)	0 (0.0)	
Dyslipidemia, *n* (%)	No	3,239 (81.8)	100 (74.1)	36 (90.0)	52 (86.7)	410 (77.5)	78 (87.6)	0.010
	Yes	721 (18.2)	35 (25.9)	4 (10.0)	8 (13.3)	119 (22.5)	11 (12.4)	
Diabetes, *n* (%)	No	3,667 (92.6)	126 (93.3)	39 (97.5)	56 (93.3)	495 (93.6)	81 (91.0)	0.716
	Yes	293 (7.4)	9 (6.7)	1 (2.5)	4 (6.7)	34 (6.4)	8 (9.0)	
Drinking, *n* (%)	Never/past	1,717 (43.4)	74 (54.8)	12 (30.0)	26 (43.3)	286 (54.1)	42 (47.2)	<0.001
	Current	2,243 (56.6)	61 (45.2)	28 (70.0)	34 (56.7)	243 (45.9)	47 (52.8)	
Smoking, *n* (%)	Never/past	3,381 (85.4)	122 (90.4)	32 (80.0)	47 (78.3)	456 (86.2)	75 (84.3)	0.178
	Current	579 (14.6)	13 (9.6)	8 (20.0)	13 (21.7)	73 (13.8)	14 (15.7)	
Walking time, min/day, *n* (%)	<60	1,424 (36.0)	38 (28.1)	14 (35.0)	26 (43.3)	201 (38.0)	27 (30.3)	0.204
	≥60	2,536 (64.0)	97 (71.9)	26 (65.0)	34 (56.7)	328 (62.0)	62 (69.7)	

*SD*, standard deviation.

^a^Analysis of variance (ANOVA) for age and chi-square tests for all variables except age. *P*-value when the missing value is excluded.

Table 2 shows baseline and follow-up vegetable intake based on the marital transition category. At baseline, intake of total vegetables, green and yellow vegetables, and light-colored vegetables was highest among the married to widowed, and lowest among the not married to married. From baseline to follow-up, vegetable intake tended to decrease in the married to widowed and married to divorced groups.

**Table 2.** Vegetable intake at baseline and follow-up

	Marital transitions					

	Consistently married	Married to widowed	Married to divorced	Not married to married	Remained not married	Missing
**Total vegetable intake, g/day,^a,b^ mean (*SD*)**						
Baseline	132.0 (79.4)	157.6 (90.1)	135.3 (72.8)	112.7 (70.8)	129.5 (88.6)	129.6 (98.3)
Follow-up	136.3 (79.8)	148.3 (80.1)	121.2 (72.3)	135.1 (80.1)	131.1 (89.2)	123.6 (82.1)

**Green and yellow vegetable intake, g/day,^a,b^ mean (*SD*)**						
Baseline	66.8 (46.2)	82.4 (56.2)	68.4 (44.5)	55.8 (39.7)	66.4 (52.0)	67.4 (60.7)
Follow-up	67.9 (46.1)	78.2 (46.0)	58.1 (46.1)	68.9 (49.4)	66.7 (54.8)	63.4 (40.6)

**Light-colored vegetable intake, g/day,^a,b^ mean (*SD*)**						
Baseline	65.2 (40.6)	75.2 (42.2)	66.8 (40.5)	56.9 (35.2)	63.1 (43.3)	62.3 (45.1)
Follow-up	68.4 (41.6)	70.1 (40.5)	63.1 (33.5)	66.1 (36.5)	64.4 (43.5)	60.2 (43.6)

*SD*, standard deviation.

^a^Vegetable intake was adjusted for total energy intake by the residual method.

^b^Missing data: *n* = 157 (3.3%) for baseline, *n* = 1,450 (30.1%) for follow-up.

Table 3 shows the results of the multivariable analysis on the association between the marital transition categories and change in vegetable intake. The results indicated that after adjusting for all covariates, married to widowed was negatively associated with change in total vegetable intake as compared to consistently married (β = −16.64, *SE* = 7.68, *P* = 0.030). In addition, married to widowed was negatively associated with change in light-colored vegetable intake (β = −11.46, *SE* = 4.33, *P* = 0.008). None of the marital transition categories were associated with change in green and yellow vegetable intake. The negative associations between married to widowed and change in total vegetable intake and light-colored vegetable intake were similar in the complete case analysis (see eTable 1). In a gender-stratified analysis, married to widowed was negatively associated with changes in total vegetable intake and light-colored vegetable intake in almost the same way for both men and women, although the associations were slightly stronger in men than in women (see eTable 2).

**Table 3.** Multivariable linear regression analysis on the association between marital transitions and change in vegetable intake

	Marital transitions^a^											

	Married to widowed vs Consistently married			Married to divorced vs Consistently married			Not married to married vs Remained not married			Remained not married vs Consistently married		
			
	β	*SE*	*P*-value	β	*SE*	*P*-value	β	*SE*	*P*-value	β	*SE*	*P*-value
**Changes in total vegetable intake**												
Age and gender adjusted	−15.73	7.66	0.040	−8.77	13.18	0.506	10.98	10.80	0.310	4.69	4.04	0.246
Multivariable adjusted^b^	−16.64	7.68	0.030	−9.39	13.23	0.478	10.92	10.84	0.314	4.93	4.81	0.306

**Changes in green and yellow vegetable intake**												
Age and gender adjusted	−4.65	4.69	0.321	−7.63	8.07	0.344	9.27	6.61	0.161	3.57	2.48	0.149
Multivariable adjusted^b^	−5.19	4.70	0.270	−7.86	8.10	0.332	9.41	6.63	0.156	4.23	2.95	0.151

**Changes in light-colored vegetable intake**												
Age and gender adjusted	−11.08	4.31	0.010	−1.14	7.39	0.877	1.71	6.07	0.778	1.12	2.27	0.622
Multivariable adjusted^b^	−11.46	4.33	0.008	−1.53	7.42	0.836	1.51	6.09	0.804	0.70	2.70	0.796

β, unstandardized regression coefficient; *SE*, standard error.

^a^All estimates are relative to “consistently married,” except that “not married to married” is relative to “remained not married.”

^b^Multivariable adjusted models included age, gender, body mass index, living arrangement, education in years, employment status, present illness (cancer, heart disease, stroke, hypertension, dyslipidemia, and diabetes), and lifestyle (drinking, smoking, and walking time).

## DISCUSSION

The present longitudinal study examined the impact of marital transitions on vegetable intake among Japanese middle-aged and older adults. The results revealed a decline in total vegetable intake and light-colored vegetable intake among participants who became widowed during the 5-year follow-up period. Our findings suggest the importance of interventions for maintaining adequate vegetable intake to prevent adverse health events due to marital transitions among middle-aged and older adults.

Our results showing that becoming widowed was associated with reduced vegetable intake support several previous longitudinal studies conducted in Western countries.^11^^–^^13^ There are several possible pathways explaining this result. First, the absence of a spouse may reduce the opportunities for eating foods that require preparation and are cooked at home, and may promote having meals in more convenient ways. These lifestyle changes related to dietary intake may cause a decrease in vegetable intake. Second, social and physiological factors associated with being married facilitate a person’s motivation to engage in healthy behaviors.^33^ This absence of spousal-facilitated social control may result in an increase in unhealthy behaviors, including low vegetable intake. Third, because those in the married to widowed group were older than those in other groups, age-related factors may be confounders. For instance, a decrease in overall dietary intake due to aging may have affected vegetable intake. However, we believe that this did not have a large effect on our results, as we confirmed that the results of further adjustment for changes in total energy intake were mostly unchanged (data not shown). However, even with statistical age adjustment, other age-related factors still need to be investigated; thus, further investigations may be necessary. Low vegetable intake has been associated with increased risk of mortality due to cardiovascular disease^18^^,^^19^ and cancer.^19^ Therefore, because reduced vegetable intake due to widowhood in middle-aged and older adults may lead to an increased risk of adverse health events, dietary counseling tailored to factor in marital transitions is considered important for preventing these events.

In the present study, transitioning from being married to widowed was significantly associated with a change in light-colored vegetable intake, and not significantly associated with green and yellow vegetable intake. Previous studies have not examined the association between marital transitions and vegetable intake separately for green and yellow vegetables and light-colored vegetables, so a comparison cannot be made; however, there are some possible explanations for the results obtained in this study. As Japanese people consume two-thirds of their vegetables from light-colored vegetables,^34^ the change in diet due to being widowed might have had a relatively larger effect on light-colored vegetable intake than on green and yellow vegetable intake. Alternatively, the need for light-colored vegetable intake might not be recognized compared to green and yellow vegetables. After marital transitions, green and yellow vegetable intake might have involved more conscious effort than consuming light-colored vegetables. As light-colored vegetables contain some essential vitamins and dietary fiber, their shortage may have adverse health effects. For instance, cabbage contains high vitamin C and K, and burdock contains high dietary fiber.^35^^,^^36^ However, although we preliminarily described nutrient intake of each marriage transition (see eTable 3), any noticeable changes in these nutrients were not observed among those who were widowed. Thus, the impact of marriage transition on nutrient intake may need further investigation. In contrast, it is also possible that a change in green and yellow vegetable intake could not be detected in the present study because of the small sample size, as point estimates showed a decreasing trend in those who experienced widowhood. Further research with large sample sizes is needed to detect these changes.

In our gender-stratified analysis, the negative association between becoming widowed and change in vegetable intake was almost similar in both men and women, although the associations were slightly stronger in men than in women. Due to the small sample size of our study, we cannot conclude definitively about gender differences, but our results are somewhat similar to those of previous longitudinal studies conducted in Western countries.^11^^–^^13^ One study in the United Kingdom reported that vegetable intake decreased in widowed men but not in widowed women.^13^ Studies in the United States indicated that decrease in vegetable intake occurred in both widowed men and women, but the effect was slightly greater in men.^11^^,^^12^ Thus, research indicates that the effect of widowhood on vegetable intake tends to be more pronounced in men than in women. Since men in heterosexual marriages are more likely to be affected by spousal social control,^37^ the absence of a spouse may increase unhealthy behaviors especially in men. Although Japan is a country in which traditional familism is valued, and housework and meal preparation are overwhelmingly performed by women,^20^^,^^21^ the effects of marital transitions on vegetable intake may show similar trends across cultures. Further studies are needed to elucidate gender differences in the effects of marital transitions on dietary habits.

The present study had several strengths. First, using 5-year longitudinal data, we were able to examine the impact of marital transitions on vegetable intake, rather than restricting the investigation to marital status at only one point in time. Second, we investigated changes in vegetable intake not only with regard to total vegetable intake but also change in the consumption of green and yellow vegetables and light-colored vegetables. Therefore, the effect of marital transitions on diet was examined in more detail.

In contrast, our study has several limitations that should be noted. First, we used a self-administered questionnaire, the FFQ, to assess vegetable intake. We could not estimate the absolute value of vegetable intake from the assessment of the frequency of intake of some vegetables included in the FFQ. However, the ability to assess relative vegetable intake allowed us to examine the association between marital transitions and vegetable intake. In addition, the FFQ we used may not be a valid assessment of vegetable intake. Since we estimated vegetable intake using only 10 items, vegetable intake may be underestimated—our estimates were small compared to the National Health and Nutrition Survey in Japan, which used dietary recording.^34^ Therefore, the external validity of our results may be low in areas where vegetables other than those we assessed are consumed in large quantities; hence, caution is needed. Second, because we had no information on participants’ income, we could not adjust for economic status in our study. Economic situations may be related to vegetable availability, which might cause residual confounding. However, we have addressed this confounding in part by considering employment status and level of education as indicators of socioeconomic status. Third, the follow-up rate in our study was moderate (70.8%), which might have caused selection bias. However, systematic error may not have occurred because there is positive evidence that people with reduced vegetable intake due to marital transitions have a higher follow-up rate. Finally, we were unable to thoroughly examine gender differences due to the small sample size, although the gender-stratified analysis showed that the reduction in vegetable intake due to becoming widowed was slightly more pronounced in men than in women. Further studies with a larger sample size are needed to investigate gender differences more comprehensively.

### Conclusions

The present study showed that becoming widowed was associated with a decrease in total vegetable intake and light-colored vegetable intake. Our findings suggest the importance of dietary counseling to prevent adverse health events following the death of a spouse.

## APPENDIX A. SUPPLEMENTARY DATA

eTable 1. Multivariable linear regression analysis on the association between marital transitions and change in vegetable intake by complete case analysis (*n* = 3,221)

eTable 2. Multivariable linear regression analysis on the association between marital transitions and change in vegetable intake by gender

eTable 3. Daily nutrient intake by the marital transitions at baseline and follow-up
