# Supplementary material for: The Impact of Marital Transitions on Vegetable Intake in Middle-aged and Older Japanese Adults: A 5-year Longitudinal Study
Source: J Epidemiol. 2022 Feb 5;32(2):89–95. doi: 10.2188/jea.JE20200343 (PMC8761567; doi:10.2188/jea.JE20200343)
Supplement: Supplementary file 1 [file je-32-089-s001.pdf]

eTable 1. Multivariable linear regression analysis on the association between marital transitions and change in vegetable intake by complete case analysis (n=3,221)

	Marital transitions [a, b]											
	Married to widowed vs. Consistently married			Married to divorced vs. Consistently married			Not married to married vs. Remained not married			Remained not-married vs. Consistently married		
	β	*SE*	*P*-value	β	*SE*	*P*-value	β	*SE*	*P*-value	β	*SE*	*P*-value
**Changes in total vegetable intake**												
Age and gender adjusted	-15.71	7.68	0.041	-8.81	13.26	0.506	11.11	10.87	0.307	4.66	4.05	0.251
Multivariable adjusted [c]	-15.53	7.79	0.046	-9.27	13.32	0.486	10.78	10.92	0.324	5.24	4.87	0.282
**Changes in green and yellow vegetable intake**												
Age and gender adjusted	-4.59	4.71	0.329	-7.69	8.13	0.345	9.46	6.67	0.156	3.52	2.49	0.157
Multivariable adjusted [c]	-4.44	4.77	0.352	-7.90	8.16	0.333	9.44	6.69	0.158	4.36	2.98	0.144
**Changes in light-colored vegetable intake**												
Age and gender adjusted	-11.11	4.31	0.010	-1.12	7.43	0.880	1.65	6.09	0.787	1.14	2.27	0.616
Multivariable adjusted [c]	-11.09	4.37	0.011	-1.38	7.48	0.854	1.34	6.13	0.827	0.88	2.73	0.747

β, unstandardized regression coefficient; *SE*, standard error.

[a] Consistently married, n=2,728; Married to widowed, n=84; Married to divorced, n=28; Not married to married, n=47; Remained not

married, n=334

[b] All estimates are relative to "consistently married," except that "not married to married" is relative to "remained not married."

[c] Multivariable adjusted models included age, gender, body mass index, living arrangement, education in years, employment status, present illness (cancer, heart disease, stroke, hypertension, dyslipidemia, and diabetes), and lifestyle (drinking, smoking, and walking time).

eTable 2. Multivariable linear regression analysis on the association between marital transitions and change in vegetable intake by gender

	Marital transitions [a]											
	Married to widowed vs. Consistently married			Married to divorced vs. Consistently married			Not married to married vs. Remained not married			Remained not married vs. Consistently married		
	β	*SE*	*P*-value	β	*SE*	*P*-value	β	*SE*	*P*-value	β	*SE*	*P*-value
Men (n=2,691)												
**Changes in total vegetable intake**												
Age adjusted	-16.25	11.26	0.149	2.23	17.22	0.897	20.48	14.29	0.152	-5.32	6.17	0.388
Multivariable adjusted [b]	-16.77	11.32	0.139	2.73	17.38	0.875	18.65	14.45	0.197	-4.22	7.31	0.564
**Changes in green and yellow vegetable intake**												
Age adjusted	-4.80	6.97	0.491	-2.44	10.66	0.819	11.46	8.84	0.195	-2.67	3.82	0.485
Multivariable adjusted [b]	-5.01	7.00	0.474	-1.22	10.75	0.910	10.29	8.93	0.249	-0.18	4.51	0.968
**Changes in light-colored vegetable intake**												
Age adjusted	-11.45	5.96	0.055	4.67	9.11	0.608	9.02	7.57	0.234	-2.66	3.27	0.416
Multivariable adjusted [b]	-11.76	6.00	0.050	3.94	9.20	0.668	8.36	7.66	0.275	-4.04	3.87	0.298
Women (n=2,122)												
**Changes in total vegetable intake**												
Age adjusted	-13.94	10.64	0.190	-22.34	20.32	0.272	6.30	16.70	0.706	11.45	5.46	0.036
Multivariable adjusted [b]	-15.06	10.71	0.160	-23.81	20.43	0.244	6.73	16.76	0.688	10.11	6.65	0.129

**Changes in green and yellow vegetable intake**												
Age adjusted	-3.44	6.44	0.593	-14.30	12.29	0.245	11.13	10.09	0.270	7.89	3.30	0.017
Multivariable adjusted [b]	-4.16	6.48	0.521	-15.15	12.35	0.220	11.98	10.13	0.237	6.70	4.03	0.096
**Changes in light-colored vegetable intake**												
Age adjusted	-10.50	6.32	0.097	-8.04	12.02	0.504	-4.83	9.91	0.626	3.57	3.23	0.270
Multivariable adjusted [b]	-10.90	6.37	0.088	-8.67	12.12	0.475	-5.26	9.97	0.598	3.40	3.94	0.388

β, unstandardized regression coefficient; SE, standard error.

[a] All estimates are relative to “consistently married,” except that “not married to married” is relative to “remained not married.”

[b] Multivariable adjusted models included age, body mass index, living arrangement, education in years, employment status, present illness (cancer, heart disease, stroke, hypertension, dyslipidemia, and diabetes), and lifestyle (drinking, smoking, and walking time).

eTable 3. Daily nutrient intake by the marital transitions at baseline and follow-up

		Marital transitions																			
		Consistently married *n*=3,960				Married to widowed *n*=135				Married to divorced *n*=40				Not married to married *n*=60				Remained not married *n*=529			
		Baseline		Follow-up		Baseline		Follow-up		Baseline		Follow-up		Baseline		Follow-up		Baseline		Follow-up	
Nutrients [a, b]	Unit	*Mean*	*SD*	*Mean*	*SD*	*Mean*	*SD*	*Mean*	*SD*	*Mean*	*SD*	*Mean*	*SD*	*Mean*	*SD*	*Mean*	*SD*	*Mean*	*SD*	*Mean*	*SD*
Energy	kcal	1,760	341	1,757	378	1,649	327	1,668	347	1,725	340	1,664	295	1,682	325	1,743	520	1,661	343	1,637	351
Protein	g	53.4	7.5	53.9	7.9	53.0	7.0	54.7	6.6	51.8	7.3	53.1	8.4	50.8	6.2	53.7	6.6	52.0	8.3	53.1	8.8
Fat	g	43.5	10.4	45.9	11.1	42.8	8.4	46.4	8.4	43.8	10.4	46.9	10.8	40.9	8.7	44.6	11.5	42.2	10.1	45.3	10.9
Carbohydrate	g	249.0	25.5	245.3	27.7	250.7	18.7	246.2	22.6	237.0	35.7	233.9	31.8	250.6	25.7	254.0	25.5	247.2	30.1	247.2	24.5
Sodium	mg	1,814	457	1,818	488	1,821	396	1,854	447	1,770	459	1,757	369	1,746	511	1,826	415	1,783	468	1,799	522
Potassium	mg	2,125	437	2,120	439	2,198	481	2,160	416	2,148	499	2,027	558	1,985	431	2,100	438	2,075	486	2,066	493
Calcium	mg	517	144	534	149	532	137	540	127	487	150	526	166	479	167	518	163	524	159	536	158
Iron	mg	7.1	1.9	7.0	1.8	7.6	1.9	7.3	1.8	6.8	1.7	6.6	1.8	6.9	2.1	7.1	1.9	6.9	1.9	6.9	2.0
Carotenes	µg	3,096	1,366	3,122	1,382	3,585	1,738	3,405	1,318	3,192	1,339	2,931	1,479	2,752	1,220	3,060	1,258	3,081	1,581	3,144	1,682
Retinol activity equivalents	µg	950	460	922	414	1079	468	970	391	971	418	780	337	857	425	860	306	921	441	907	442
Vitamin D	µg	7	3	7	3	8	3	7	3	7	3	7	3	6	3	7	4	7	3	7	4
Vitamin E	mg	8.0	1.9	8.2	2.0	8.4	1.8	8.5	1.8	8.2	1.7	8.1	2.2	7.4	1.6	8.3	2.1	7.8	2.2	8.1	2.2
Vitamin B1	mg	0.65	0.08	0.66	0.09	0.64	0.07	0.66	0.08	0.65	0.09	0.65	0.06	0.64	0.08	0.67	0.10	0.63	0.09	0.64	0.09

Vitamin B2	mg	1.08	0.26	1.09	0.26	1.12	0.24	1.12	0.24	1.06	0.26	1.08	0.26	1.03	0.22	1.06	0.27	1.08	0.25	1.09	0.27
Folate	μg	338	107	333	103	375	117	348	99	335	84	305	87	317	89	330	102	334	110	325	108
Vitamin C	mg	99	37	99	36	110	36	107	39	98	32	94	30	93	38	97	36	99	39	97	39
Total dietary fiber	g	10.9	3.1	11.1	3.2	12.0	3.4	12.2	3.4	11.0	3.3	10.7	2.8	10.4	3.0	11.5	3.6	10.9	3.4	11.3	3.6
Insoluble dietary fiber	g	7.9	2.2	8.1	2.3	8.7	2.4	8.8	2.5	8.0	2.2	7.9	2.0	7.5	2.0	8.3	2.9	7.9	2.4	8.2	2.6
Soluble dietary fiber	g	2.0	0.6	2.0	0.7	2.2	0.6	2.3	0.7	2.0	0.7	2.0	0.5	1. 9	0.6	2.1	0.7	2.0	0.7	2.1	0.7
Cholesterol	mg	236	63	238	65	237	57	249	60	228	58	242	67	230	67	237	60	228	65	231	69
Saturated fatty acids	g	11.19	2.60	11.46	2.65	11.02	2.29	11.36	2.17	10.99	2.46	11.52	3.30	10.47	1.82	10.56	2.21	11.07	2.62	11.21	2.56
Monounsaturated fatty acids	g	15.98	3.72	16.66	3.94	15.77	2.98	16.65	3.35	16.13	3.56	16.74	4.17	15.45	3.28	16.67	3.91	15.20	3.65	16.25	3.86
Polyunsaturated fatty acids	g	12.86	3.08	13.10	3.18	13.06	2.76	13.31	3.13	12.57	2.87	12.73	3.27	12.07	2.83	12.98	3.33	12.29	3.12	12.75	3.13
n-6 Polyunsaturated fatty acids	mg	10,873	2,753	11,122	2,796	10,994	2,577	11,325	2,698	10,807	2,745	10,998	2,905	10,307	2,714	10,812	2,939	10,484	2,840	10,853	2,601
n-3 Polyunsaturated fatty acids	mg	2,193	510	2,225	532	2,238	427	2,239	427	2,209	541	2,225	633	2,079	434	2,238	518	2,096	528	2,181	588
n-3 Highly-unsaturated fatty acids	mg	693	288	60	117	732	248	52	98	660	262	113	157	609	235	41	88	658	314	56	103

SD, standard deviation.

[a] Missing data: n =157 (3.3%) for baseline, n=1,450 (30.1%) for follow-up.

[b] Daily nutrient intake was adjusted for total energy intake by the residual method.
